# Community engagement and clinical trial diversity: Navigating barriers and co-designing solutions—A report from the “Health Equity through Diversity” seminar series

**DOI:** 10.1371/journal.pone.0281940

**Published:** 2023-02-16

**Authors:** Luiza Reopell, Timiya S. Nolan, Darrell M. Gray, Amaris Williams, LaPrincess C. Brewer, Ashley Leak Bryant, Gerren Wilson, Emily Williams, Clarence Jones, Alicia McKoy, Jeff Grever, Adam Soliman, Jna Baez, Saira Nawaz, Daniel M. Walker, Faith Metlock, Lauren Zappe, John Gregory, Joshua J. Joseph

**Affiliations:** 1 The Ohio State University College of Medicine, Columbus, OH, United States of America; 2 The Ohio State University College of Nursing, Columbus, OH, United States of America; 3 The Ohio State University James Center for Cancer Health Equity, Columbus, OH, United States of America; 4 Department of Cardiovascular Medicine, Mayo Clinic College of Medicine, Rochester, MN, United States of America; 5 The University of North Carolina at Chapel Hill School of Nursing, Chapel Hill, NC, United States of America; 6 Genentech Inc., San Francisco, CA, United States of America; 7 Franklin University, Columbus, OH, United States of America; 8 Hue-Man Partnership, Minneapolis, MN, United States of America; 9 The Ohio State University College of Public Health, Columbus, OH, United States of America; 10 The African American Male Wellness Agency, National Center for Urban Solutions, Columbus, OH, United States of America; Virginia Commonwealth University, UNITED STATES

## Abstract

**Introduction:**

In recent years, there has been increasing awareness of the lack of diversity among clinical trial participants. Equitable representation is key when testing novel therapeutic and non-therapeutic interventions to ensure safety and efficacy across populations. Unfortunately, in the United States (US), racial and ethnic minority populations continue to be underrepresented in clinical trials compared to their White counterparts.

**Methods:**

Two webinars in a four-part series, titled “Health Equity through Diversity,” were held to discuss solutions for advancing health equity through diversifying clinical trials and addressing medical mistrust in communities. Each webinar was 1.5 hours long, beginning with panelist discussions followed by breakout rooms where moderators led discussions related to health equity and scribes recorded each room’s conversations. The diverse groups of panelists included community members, civic representatives, clinician-scientists, and biopharmaceutical representatives. Scribe notes from discussions were collected and thematically analyzed to uncover the central themes.

**Results:**

The first two webinars were attended by 242 and 205 individuals, respectively. The attendees represented 25 US states, four countries outside the US, and shared various backgrounds including community members, clinician/researchers, government organizations, biotechnology/biopharmaceutical professionals, and others. Barriers to clinical trial participation are broadly grouped into the themes of access, awareness, discrimination and racism, and workforce diversity. Participants noted that innovative, community-engaged, co-designed solutions are essential.

**Conclusions:**

Despite racial and ethnic minority groups making up nearly half of the US population, underrepresentation in clinical trials remains a critical challenge. The community engaged co-developed solutions detailed in this report to address access, awareness, discrimination and racism, and workforce diversity are critical to advancing clinical trial diversity.

## Introduction

Efforts to engage diverse populations in clinical research are critical to scientific progress and advancing health equity in the United States (US) [1]. Although one-fifth of new drugs demonstrate differences in exposure and/or response across racial and ethnic groups, Black and Hispanic/Latinx populations are significantly underrepresented in clinical research studies. This omission has been ongoing specifically in endocrine, diabetes, cancer, and cardiovascular disease research [2–7]. This is particularly troubling because common diseases with a high prevalence and mortality in Black populations, such as hypertension and heart failure, have responded differently to medications in racial/ethnic groups [8, 9].

Furthermore, the growth of racial and ethnic minority populations in the US is not reflected in clinical trials. The Federal Drug Administration (FDA) reports found that African Americans are 13% of the US population, but only make up 9% of clinical trial participants [10]. Black populations represented 16% of clinical trial participants in medication clinical trials at US sites from 2015–2019, although a preponderance of those participants were in infectious disease and psychiatry clinical trials, with a profound underrepresentation in cardiovascular and cancer clinical trials [3, 4]. This could be due to organizations recruiting for psychiatry studies having a higher prevalence of un- and under-insured patients, of which there is a higher prevalence in racial/ethnic minority patients [11]. Infectious disease trials are less likely to take place in private health care organizations, and African American populations are more likely to receive care at fee-for-service and public organizations [12]. In the US, similar data show that 15% of clinical trial participants are Hispanic or Latinx, with overrepresentation in gynecology studies but underrepresentation across many other areas including cardiovascular disease and cancer [3, 4]. There is a higher prevalence of African American and Hispanic/Latino residents and physicians within the obstetrics and gynecology specialty as compared to other specialties, which could explain better recruitment into these types of trials [13].

Medications and interventions are routinely tested for safety, but with low inclusion of racial and ethnic minorities in clinical trials, safety data could be lacking for racial and ethnic minorities [7]. Additionally, exclusion of racial/ethnic minorities in clinical trials may exacerbate health disparities due to potential differences in efficacy, prescribing, or patient comfort and trust in a novel medication [14, 15].

A common misconception around low accrual of racial/ethnic minorities to clinical trials is that they are uninterested in clinical trials; however, studies show this is not the case [16]. A literature review examining 70,000 participants’ decisions to enroll in 20 health research studies including interviews, drug treatment, and surgical trials, found that Black and Hispanic individuals were just as willing to participate in clinical trials as non-Hispanic Whites [16]. This suggests that the lack of minority participation in clinical trials may not be due to lack of interest, but may be caused by barriers preventing participation more common in racial/ethnic minority populations, ineffective strategies to address these barriers, and challenges with retention.

Barriers to clinical trial participation described in the extant literature include mistrust, lack of comfort with the research process, lack of information, time and resource constraints, and lack of awareness [4, 5, 17, 18]. One solution is enhancing the diversity within the clinical trial workforce, including principal investigators and clinical research coordinators. A long standing lack of underrepresented minority principal investigators exists due to a number of factors, such as a lack of grant funding for studies pertaining to community-based research, population research, and racial/ethnic disparities [19, 20]. However, new challenges as a result of the COVID-19 pandemic have illuminated existing barriers and created additional barriers to clinical trial participation [21, 22]. Given our commitment to community engagement as a strategy to co-design equitable solutions [23, 24], we applied that same lens to clinical trial diversity by establishing partnerships with community members, The National African American Male Wellness Agency, pharmaceutical companies, government organizations, and academic institutions to collaborate on an interactive, community-facing, live webinar series focused on “Health Equity Through Diversity: From Communities to Clinics to Clinical Trials” [1]. Here we will discuss how we combined the information we learned from the Webinar to identify barriers, and elucidate actionable solutions to increase and promote clinical trial diversity.

## Methods

Two “Health Equity through Diversity” webinars aimed to co-create solutions that both advance health equity through diversifying clinical trials and the clinical trial and healthcare workforce and heal medical mistrust in communities through meaningful partnerships and community engagement. The first and second webinars were entitled “Clinical Trials and Underrepresented Minorities: Mistrust, Misconceptions, Missed Opportunities and Moving Forward to Enhance Diversity,” held in April 2021 and “Community Engagement: Strategies for Cultivating and Sustaining Meaningful Partnerships,” held in June 2021. Webinar information was advertised on social media outlets including LinkedIn, Twitter, Instagram and Facebook to reach a wide array of audiences and to recruit diverse attendees. Each 1.5 hour webinar began with intentionally diverse panels featuring scientists, government public health officials, biopharmaceutical representatives, community stakeholders, and community members who characterized a themed, focal area (Table 1). To identify stakeholders, the Health Equity through Diversity oversight committee reviewed the literature relevant to the themed focal areas and sought out leaders in these spaces. Committee members then utilized digital media strategies and their own professional networks to contact and recruit these stakeholders. We collaborated with a community partner, The African American Male Wellness Agency, who has a near 20-year history of engaging racial and ethnic minority groups in the Midwest, to determine webinar themes and related questions. Each individual webinar began with an introduction by one of the hosts (DMG, JJJ, or TSN), who introduced each speaker with a brief background. Speakers then gave 5-10-minute presentations that summarized the theme within the context of their work and lived experiences, which was followed by a brief question and answer session. After all the speakers had presented, the audience was apprised of breakout session ground rules and questions to guide the discussion.

**Table 1:** Health Equity through Diversity webinar series–*speakers and moderators*.

	Webinar #1: Clinical trials and Underrepresented Minorities: Mistrust, Misconceptions, Missed Opportunities and Moving Forward to Enhance Diversity	Webinar #1: Clinical trials and Underrepresented Minorities: Mistrust, Misconceptions, Missed Opportunities and Moving Forward to Enhance Diversity
Civic Representatives	Congresswoman Joyce Beatty, OH-3, Chair of the Congressional Black Caucus	Torian Easterling, MD, MDPH, Deputy Commissioner and Chief Health Equity Officer, New York City Department of Health and Mental Hygiene
		Joe Mazzola, MPA, Health Commissioner Franklin County Public Health, OH
Community Members	Matt Barnes, NBC4 Columbus News Anchor	John Drummond, Senior Chief of Staff, New Order National Human Rights Organization
	Oyama Hall, City of Columbus Network Engineer	
Community Partners		Clarence Jones, Med, CPH, CHW, Co-Founder the Hue-MAN Partnership Project
Clinician-Scientists	Ashley Leak Bryant, PhD, RN-BC, OCN, Associate Professor of Nursing, University of North Carolina Chapel Hill School of Nursing	LaPrincess Brewer, MD, MPH, Cardiologist, Assistant Professor of Medicine, Mayo Clinic
Biopharmaceutical Representatives	Gerren Wilson, PharmD, Head of Inclusion, Strategy and Partnering in the Chief Diversity Office at Genentech	Bert Bruce, MBA, Regional President North America, Rare Disease at Pfizer, Inc.
Moderators	Timiya Nolan, PhD, APRN-CNP, The Ohio State University College of Nursing, The Ohio State University James Center for Cancer Health Equity	Timiya Nolan, PhD, APRN-CNP, The Ohio State University College of Nursing, The Ohio State University James Center for Cancer Health Equity
	Joshua Joseph, MD, MPH, Assistant Professor of Medicine, The Ohio State University College of Medicine	Melissa Gonzales, PhD, External Partnering, Chief Diversity Office at Genentech
	Brent Walters, Vice President of the National African American Male Wellness Agency	Autumn Glover, MCRP, MPA, President, Partners Achieving Community Transformation (PACT), Co-Chair, Health Equity Steering Committee at The Ohio State Wexner Medical Center
		Marlon Platt, Executive Director at the National African American Male Wellness Agency
		Darrell M. Gray, II, MD, MPH, FACG
		Joshua Joseph, MD, MPH, Assistant Professor of Medicine, The Ohio State University College of Medicine
Sociodemographic Breakdown		
	Event Moderators and Speakers	Breakout Room Moderators and Scribes
African American	89%	70%
Non-Hispanic White	5.5%	21%
Hispanic/Latino	5.5%	6.5%
Asian	0%	2.5%

Each breakout session, led by a moderator with a scribe, lasted 15–20 minutes. The questions discussed in the breakout rooms were the learning objectives for each webinar. The questions for webinars 1 and 2, respectively were: 1) “Currently with all the great studies and science, only 5% of clinical trial participants are Black or African American and only 1% of clinical trial participants are Hispanic or Latino Origin. What are the missing pieces? What are potential solutions to increase these numbers?”; and 2) “How can we drive meaningful partnerships to advance health among community members and stakeholders with healthcare orgs, industry, and government?” (Table 2).

**Table 2:** Health Equity through Diversity webinar series–*breakout room prompts and questions*.

Health Equity through Diversity Webinar Series–*Breakout Room Prompts and Questions*	Webinar #1: Clinical trials and Underrepresented Minorities: Mistrust, Misconceptions, Missed Opportunities and Moving Forward to Enhance Diversity	Webinar #2: Community Engagement: Strategies for Cultivating and Sustaining Meaningful Partnerships
Prompt	Currently with all the great studies and science, only 5% of clinical trial participants are Black or African American and only 1% of clinical trial participants are Hispanic or Latino Origin.	How can we drive meaningful partnerships to advance health among community members and stakeholders with health care organizations, industry and government?
Question 1	What are the missing pieces?	What does meaningful partnership mean to you?
Question 2	What are potential solutions to increase these numbers?	Have you engaged in any meaningful partnerships in the last 5 years?
Question 3	N/A	What were the impact and outcomes from the partnership?

The audience was intentionally divided into breakout session groups to ensure a diversity of academic, community member, clinician researcher, biotechnology and biopharmaceutical representatives, and employees or representatives of government organizations in each group, so that all perspectives would be included and documented in the conversation. There were 20 breakout rooms in the first webinar session, and 16 in the second. Each breakout room had the moderator, scribe, and approximately 12 attendees in the first seminar and 13 in the second seminar. Scribed notes have been found to yield equally rich and comprehensive data as transcribed notes while being more economical [25]. Due to the nature of wanting to keep the webinars free and accessible to all, using qualified volunteers as scribes was the optimal choice for our purposes. Following each webinar, scribed notes from breakout rooms were collated by LR and AW. The notes from the scribes were not verbatim, but rather notes from college or graduate students or faculty members in the healthcare sciences that were all given the same instructions to capture statements and sentiments of the discussion (S1 File). To analyze the resulting data, we utilized thematic analysis for its ability to provide a complex and detailed report of the collected data [26]. We wanted to identify and categorize the common themes expressed throughout the many breakout sessions [27]. Codes were refined and finalized through an iterative process, which included 1) LR and AW independently reviewing and coming to consensus on an initial coding schema and 2) all other co-authors on the manuscript (including both qualitative and community outreach and engagement experts) reviewing and refining the coding schema until 100% consensus was attained. The schema was then finalized after attaining validation amongst stakeholders representing scientists, government public health officials, biopharmaceutical representatives, community stakeholders, and community members.

The aforementioned methods were applied to answer the research question: What are the novel and existing strategies to advance clinical trial diversity? This project used existing data collected for non-research purposes, with no identifiers linking human participants to the data. The Ohio State University Biomedical Sciences Institutional Review Board and Office of Responsible Research Practices determined that the project does not meet the federal definition of human subjects’ research requiring review.

## Results

**Fig 1:**
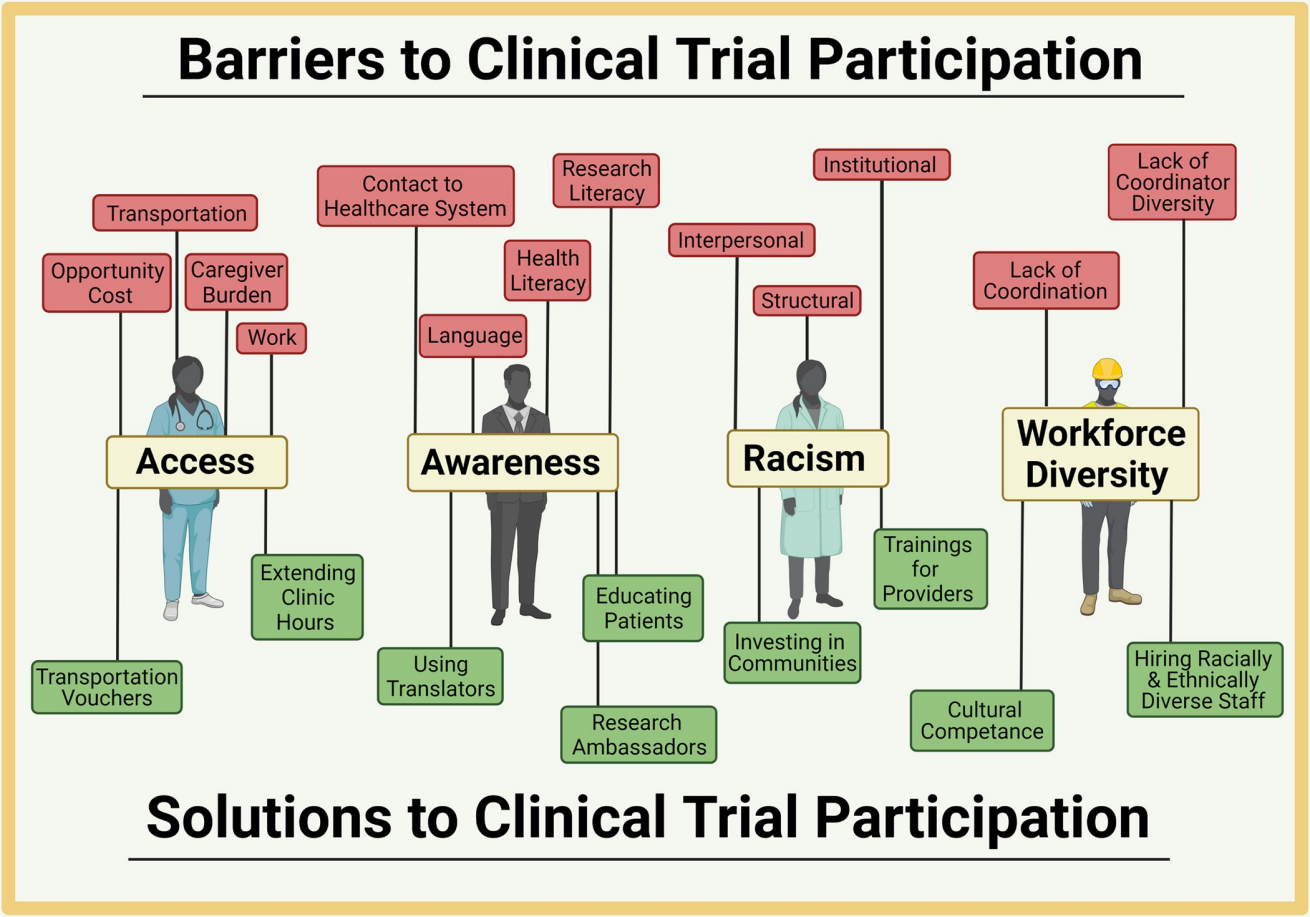
Barriers and solutions to clinical trial participation. This figure displays the four major themes explored in this paper and the barriers and solutions associated with each theme.

Two-hundred and forty-two individuals attended the first webinar and 205 attendees participated in the second webinar (Table 3) from 25 states across the US, and 4 countries outside the US. At webinars #1 and #2, attendee percentages were: 17% and 16% for community members, 30% and 25% for clinicians/researchers, 11% and 12% for government organizations, 13% and 11% for biotechnology/biopharmaceutical participants and 29% and 36% for other participants, respectively. Multiple universities, medical centers, biopharmaceutical companies, government organizations, and community stakeholders and members were represented. The attendees and panelists provided information relative to novel and existing strategies to advance clinical trial diversity. From the panel presentations and breakout room discussions, four broad themes of barriers and related solutions emerged around 1) access; 2) awareness; 3) discrimination and racism; and 4) workforce diversity (Fig 1). Specific barriers and solutions are presented in Table 4.

**Table 3:** Demographics of webinar #1 and #2 participants.

	Webinar #1: Clinical trials and Underrepresented Minorities: Mistrust, Misconceptions, Missed Opportunities and Moving Forward to Enhance Diversity	Webinar #2: Community Engagement: Strategies for Cultivating and Sustaining Meaningful Partnerships
Registrants	417	279
Attendees	242	205
US Location of Attendees US	25 US States	23 US States
International Location of Attendees	France, Great Britain	Iceland, Brazil
Attendee Background		
Community Member	41 (17%)	33 (16%)
Clinician/Researcher	73 (30%)	51 (25%)
Employee/Representative of Government Organization	27 (11%)	25 (12%)
Biotechnology/Biopharmaceutical Organization	31 (13%)	23 (11%)
Other	70 (29%)	74 (36%)

**Table 4:** Barriers and solutions in the conference and current literature.

**Barrier–Access**	**Conference**	**Current literature**
	• Individuals are unwilling or unable to participate in trials due to lack of childcare and elder care during visits • Office hours of clinics and labs are the same as many jobs, so it is difficult to get off work • Cost of healthcare prevents people from participating in primary healthcare which is a major site of recruitment	• Research sites are far from the participants [30] • Transportation is difficult for people, especially when research sites are in unfamiliar locations [29] • Minority populations engage in more caregiving, meaning they don’t have time to devote to trials [31] • Minority groups are overrepresented in service occupations [34] • Minority groups have a more difficult time paying for healthcare services [28] • Minority groups consider opportunity costs of decisions more than White groups [35]
**Solutions—Access**	**Conference**	**Current Literature**
	• Include incentives and compensation when designing research trial budget • There should be a national standard for paying for parking, childcare and missed time at work • Hold research visits outside of business hours so people can attend more easily	• 3 different types of payment for research participants–reimbursement, compensation and incentives; all should be given so that participants are not worse off [36]
**Barrier—Awareness**	• Research staff are not recruiting minority populations; they are not going to the areas with large numbers of minority populations and not making an effort to reach them or contact them • Clinicians themselves may not have the resources to inform their patients about research studies • People unaware of search engines for research trials or how to locate trials	• Minority populations are less likely to access primary healthcare services where they would learn about clinical trials; they are more likely to utilize EDs [37] • Some studies exclude non-English speakers in their eligibility criteria [39] • Recruitment materials must be in the native language of participants [38] • Ethnic/minority groups have greater challenges with health literacy [41, 42] and comprehension of the consent process [43]
**Solution–Awareness**	**Conference**	**Current Literature**
	• Make recruitment materials relevant and digestible to minority populations	• Utilize various channels to promote and advertise recruitment opportunities to reach everyone [44] • Have research ambassadors to inform potential participants about trials [45, 46]• Engage in Community-based participatory research (CBPR) to co-develop recruitment strategies with the community [47]
**Barrier—Racism**	**Conference**	**Current Literature**
	• Individuals are skeptical of clinical trials, especially ones involving medication, due to fear of being experiments	• Racial/ethnic minority groups do not trust the medical institution due to a history of racist abuse [48–57] • Minority populations still face racism and discrimination from the medical field today so they do not want to participate [58–60]
**Solution—Racism**	**Conference**	**Current Literature**
	• Investigators should engage with each participant individually to share their values and connection to the community • Educate participants on the medications or interventions used • PIs should outline how they monitor participants’ safety throughout a trial • Increase awareness on the researcher side of undocumented minority groups’ fears of working with government institutions • Educate the workforce about historical trauma minority groups may have so that they approach with cultural humility • Place specific targets of amount of non-White participants a study must have	• Engage community to understand their needs and values [1, 24] • Inform groups of how research benefits them [61]• Educate the community by working with local, trusted organizations [62]• Provide communities with the results of studies that are directly benefitting them [63]• Publish more work on how to address racism in all forms, and how racism affects health [64–66]
**Barrier–Workforce Diversity**	**Conference**	**Current Literature**
	• Some people do not feel comfortable working with healthcare workers of different race or sex • Language barriers often exist between participant and research staff• Lack of cultural competency from research staff	• Lack of diversity of clinical trial coordinators [5, 20, 70] • Lack of concordance between research staff and participant population leads to less recruitment of those groups [69, 71, 72] • Not having research staff who can speak the same language as participants [28] • Not enough diversity of investigators or scientists [73] • Research in areas more commonly chosen by minority investigators do not receive as much funding [74]
**Solution–Workforce Diversity**	**Conference**	**Current Literature**
	• Increase the amount of minority PCPs and investigators, and have more diversity in the clinical workforce generally	• Researchers should establish relationships with providers and clinics in communities of interest [63] • Create programs that support minority and underrepresented investigators in research [75]

Under the theme of access, attendees highlighted the barriers overrepresented in minority populations, such as having to serve as caregivers to family members and not having the time for trials, working in service jobs where they are unable to get time off work, and the burdensome cost of healthcare. Suggested solutions to these challenges included allocating a budget for compensation to participants for taking time off work, providing childcare and eldercare, and extending hours for research visits beyond traditional business hours.

In the discussions on the theme of awareness, the challenges of minority groups not being the focus of recruitment strategies, language barriers preventing minorities from learning about trials, and the lack of knowledge of research search engines were identified as critical areas. Attendees stressed the importance of tailoring recruitment efforts and materials to different racial/ethnic populations so that they are in languages they understand and being advertised in forums with which they have contact.

Discrimination and racism were mentioned in relation to almost all themes, but participants in the webinar series especially noted that racial/ethnic minority populations have a history of trauma and abuse associated with the medical field, leading to distrust in the present day and a fear of being used in experiments. They offered many solutions to combat this, including educating the community about the benefits of trials, and also educating the medical workforce on the racism and discriminations experienced by these populations.

The final broad theme discussed was the severe lack of diversity in the research trial workforce, which exacerbates language barriers and decreases cultural competency. Conversations that emerged from the webinar stressed the need to improve the diversity of the workforce in order to increase participant diversity.

## Discussion

The “Health Equity through Diversity: From Communities to Clinics to Clinical Trials” webinar series is a novel strategy to advance health equity through diversifying clinical trials and the clinical trial and healthcare workforce and to address medical mistrust in communities. The “Clinical Trials and Underrepresented Minorities: Mistrust, Misconceptions, Missed Opportunities and Moving Forward to Enhance Diversity” and “Community Engagement: Strategies for Cultivating and Sustaining Meaningful Partnerships” webinars illuminated novel and existing barriers and strategies to advance clinical trial diversity. The diverse group of attendees representing broad swaths of important stakeholders led to inclusive and comprehensive discussions on issues surrounding clinical trial diversity. The diversity of participants was a major strength in the co-development of solutions in the community-engaged framework.

### Access as a barrier

Limited access to healthcare is a major barrier to clinical trial recruitment that disproportionately impacts racial/ethnic minorities [28]. Often, research sites are not located in communities with a high prevalence of racial/ethnic minorities [29]. Thus, participating in research studies may be more difficult due to limited access to affordable and convenient transportation or anxiety from traveling to an unfamiliar location [30]. In our own clinical trial experience, we have seen how limited access to efficient and affordable transportation can make it difficult for participants living at a greater distance to make it to their appointments.

Another factor impeding access to research is the burden disproportionately faced by caregivers from racial/ethnic minority populations. It may be difficult for individuals who care for their family members to take time away from these duties for research visits. Rote et al. found that non-Latino Black, Mexican-origin, and other Latinx individuals were engaged in more frequent and time-intensive elder care compared to non-Latino Whites [31–33]. If these populations are more often the ones needing to care for family members in the home, they may have less time to engage in clinical trials, especially if they have no additional assistance in caretaking duties.

According to the Bureau of Labor Statistics, individuals who identify as Black/African-American or Hispanic/Latinx are the most represented group in service occupations, as well as production, transportation, and material occupations [34]. It is traditionally harder to get paid time off within these occupations, with many employers not offering it at all. During the webinar series, attendees discussed how office hours for clinics and labs that conduct research are typically open during the day, only operating during traditional 9–5 hours, making it difficult or impossible for those in service occupations to attend research visits without taking time off from work. In our studies, we have had many individuals who were interested in participating, but had to decline because they could not afford to take time away from their jobs. This gap can be described as the opportunity cost of research participation. While our literature review did not come across any studies focused on the impact of opportunity costs on racial/ethnic minority groups’ decision to participate in research studies, some studies have shown that minority groups tend to consider opportunity costs more than White populations [35]. Given the lack of current research on opportunity costs and clinical research participation decision-making across racial/ethnic groups, this topic is an important area for future research.

### Access solutions

Webinar series attendees discussed solutions to address access-related barriers to study participation, concluding that the inclusion of incentives or compensation as part of the research budget at the institutional level might help to resolve some of these issues. Research institutions and universities should ensure funds are allocated to research studies so that there is always a budget to provide compensation to participants for their time. Paying for or providing childcare, eldercare, and compensating for missed time at work would incentivize many people to participate, as those burdens would be eliminated. Some groups mentioned the importance of making this approach not just institutional, but a national standard for all trials. Additionally, extending clinic hours and having visits outside of traditional office hours would allow individuals who work during the day to participate in trials.

Providing payment for research trial participation is essential to increasing trial diversity because it reduces burdens on individuals in lower socio-economic levels. Minority populations are overrepresented in lower socioeconomic groups, therefore increasing financial remuneration may increase racial/ethnic diversity. Bierer et.al outline the different forms of payment for trials–reimbursement, compensation, and incentives. Patients must be reimbursed for all study-related costs, and compensation for their time and effort in a study is also important. Additionally, budgeting for incentives to participants further increases recruitment of individuals and is a useful strategy for increasing clinical trial diversity [36].

The clinical trialists in the webinar series noted that research-related care should be at no cost to the participant or their insurance. Compensation is provided directly to participants at the time of the research visit to limit the barrier of opportunity cost. Many researchers also suggested providing transportation vouchers to address transportation costs.

Academic medical centers have shifted toward placing research clinics in community-based ambulatory primary care office sites to assist with recruitment, making studies more accessible to potential participants’ neighborhoods. Lastly, some research teams noted success with early morning visits prior to the start of the work-day and evening and weekend research-related activities, thus improving accessibility and retention.

### Awareness as a barrier

Awareness is a major barrier to clinical trial participation. Many clinical trial participants find out about clinical trials and research through their physician or doctor’s office. Compared to 7% of White individuals, 23% of Black individuals reported having trouble paying for healthcare services, and racial/ethnic minority populations are less likely to have health insurance [28]. Additionally, racial/ethnic minority groups are more likely to utilize emergency departments for concerns that could be addressed through primary care, and are more likely not to have a primary care provider, meaning many individuals within these minority groups are missing the opportunity to learn about relevant clinical trials available to them [37]. Both of these factors are barriers to the recruitment of racial/ethnic minority groups.

Language is a barrier to clinical trial participation for non-English speaking US populations, which are predominantly racial/ethnic minority groups. In order to communicate and recruit individuals who do not speak English, studies must have interpreters to properly communicate about the study, recruitment, and consent. Materials also need to be translated to the participants’ native language, and not all studies offer this option [38]. Additionally, some studies exclude non-English speakers in their study inclusion/exclusion criteria [39], missing out on potential opportunities for increasing diversity in clinical trials. Even individuals with English as a second language may have challenges in functional health literacy, defined as “the ability to read, understand, and act on health information,” which is a major concern for American patients [40]. Individuals with low health literacy face significant challenges in understanding medical diagnoses, how to take prescription medications, and instructions on how to manage medical conditions. Racial/ethnic minority populations have lower health literacy scores compared to White populations, especially Hispanic/Latinx and Black populations [41], Individuals with lower health literacy are more likely to use emergency health services, and less likely to use preventive primary care services than those with high health literacy, which, as noted previously, is a primary site of clinical trial access and recruitment [42]. For patients utilizing primary care services, primary care providers may need to spend extra time explaining things carefully to patients, leaving little to no time to focus on clinical trial recruitment in visits that are already time constrained.

In addition to health literacy, research literacy may be a barrier to clinical trial diversity. Minority populations may not know how or where to find studies in which to participate, and once they do they may not understand all parts of the process. There are many search engines available to help individuals locate studies that are open for enrollment on the local and national level, such as ResearchMatch (researchmatch.org) or clinicaltrials.gov, but these are not generally well-known. Additionally, once participants have found a research study in which to participate, they may encounter further challenges in understanding the requirements or consent process, which could deter them from participating. One study examined comprehension of consent documents among different racial/ethnic groups [43]. African American, Hispanic, and Asian/Pacific Islander populations had lower comprehension scores than the White group [43]. Understanding what is stated in the consent form for a study is essential for the safety of individuals participating in research, however it also creates another barrier for racial/ethnic minority populations’ inclusion in clinical trials.

### Awareness solutions

Many strategies exist to address awareness of research in racial/ethnic minority communities and engage a greater proportion of these populations. In the webinar series, it was noted that recruitment materials must be both relevant and accessible to racial/ethnic minority populations. One study found that presenting culturally-tailored information about studies and disseminating this information consistently and through multiple channels increased recruitment of African American individuals into a cancer screening trial [44]. Utilizing research ambassadors (community members who have participated in research) is another method to increase awareness for minority populations, as non-White patients are more likely to obtain information about research trials from other patients than from physicians or the internet [45]. The Ohio State University Center for Clinical and Translational Sciences and other academic institutions have developed the “Community Scientist Academy”–a 6-week boot camp to increase community members’ and stakeholders’ awareness and understanding of research and to increase engagement throughout the entire translational research process. Participants from this program are equipped to go out into their communities and be ambassadors for clinical trial participation [46].

Increasing knowledge of research trials in racial/ethnic minority populations requires research staff to engage with the communities of interest. Community-based participatory research (CBPR) is an effective strategy for recruiting minority populations because it empowers communities to become involved in the entire research process [47]. CBPR gets community stakeholders involved from the start, thus allowing the research team to co-develop recruitment strategies tailored to the community of focus [47]. CBPR, community-engagement, and academic-community-government partnerships are key solutions in the armamentarium to increase clinical trial diversity, but take intentional efforts from invested institutions, researchers, and communities [1, 23, 24, 47].

### Racism in research

Racism in its many forms is a critical factor inhibiting clinical trial diversity. There are various complex issues surrounding perceived, interpersonal, institutional, and structural racism that serve as major obstacles to recruitment of minority groups in research (Table 5).

**Table 5:** Definitions for types of racism [83].

Type of Racism	Definition	Examples applicable to clinical trial participation
Internalized Racism	Beliefs individuals have surrounding race and racism. These are private thoughts individuals have and can include prejudice against other races, belief of your race as superior, feelings of inferiority based on your race, etc.	Healthcare and research staff may hold beliefs that minority individuals do not want to be a part of research, or are not reliable so they do not recruit them.
Interpersonal Racism	Overt racism that manifests when individuals interact and their internalized racism is vocalized or expressed through words or violence	Minority individuals have negative interactions with healthcare and research staff, experiencing racism in those settings, which leads to them not wanting to participate in the system in any form.
Institutional Racism	Racism that occurs in institutions that privileges the white population and produces disadvantages and inequalities for people of color based on the institutions practices and policies	There has been a history of medical abuses against racial/ethnic minority populations perpetuated by academia and government, which leads to a lack of trust from these groups, preventing clinical trial participation
Structural Racism	This is racism at the societal level. Different factors accumulate to disproportionately and systematically give privilege to the white population, and create disadvantages for people of color at all levels of society	Structural racism is the driver of social determinants of health e.g. transportation, housing, education, income, which all impact clinical trial participation

A major barrier to recruiting minorities to clinical research trials is lack of trust [24]. This issue is one of the most studied barriers to diverse clinical trial participation, as it is arguably the most significant [48]. Mistrust in the medical and scientific communities is directly linked to the issue of anti-racial/ethnic minority group racism [49]. All forms of racism, including structural racism, have devastating health consequences and are at the root of many of the US health disparities, whether directly linked to medical outcomes or by creating medical mistrust [49].

There is an extensive history of racial/ethnic minority groups being abused, exploited, and endangered in the medical field and beyond. One of the most commonly cited examples is the Tuskegee Syphilis Study on African American Men [50]. This may be one of the most well-known instances, but there are many others that further solidify the mistrust minority populations have in the medical field–The Havasupai diabetes project in the Native American population [51], the forced sterilization of Native American women [52], Latinx women [53], and inmates [54] and the sexually transmitted disease experiments in Guatemala [55]. Many of these instances, among others, have been well documented in *Medical Apartheid* by Harriet A. Washington [56]. These events have led to a combination of mistrust and fear of healthcare personnel and the healthcare system. Minority groups may feel that by participating in a clinical trial, they will be unethically experimented on for new medical interventions [28, 57]. The clinical trial investigators on the webinars noted much greater resistance for studies involving medications vs. non-medication intervention, particularly if the medication was for prevention. Investigators noted participants were concerned about why they were being chosen for trials when contacted by research teams and specifically questioning why they were being recruited. The history of mistreatment faced by racial/ethnic minority groups may lead some to assume the worst when being approached about research.

While historical events play a significant role in increasing mistrust of the healthcare field in Black populations, there is also the issue of present-day discrimination and racism in healthcare [38, 58, 59]. This racism can be manifested as overt, interpersonal racism in interactions with healthcare staff [58, 59]. Additionally, there is extensive evidence that racial/ethnic minority populations in general and especially Black populations have worse health outcomes due in part to receiving lower quality of care [60]. The combination of historical and present-day racism and unethical abuses in research is a major barrier to gaining trust and clinical trial recruitment.

### Solutions to racism in research

An effective strategy for gaining the trust of racial/ethnic minority populations as a researcher is to engage with the community and understand their needs so that research studies address the needs and values of the community [1, 24]. Oftentimes, racial/ethnic minority groups feel more motivated and willing to participate in a study if they believe it will directly benefit them or their friends and relatives [61]. Some investigators on the webinars noted they engage with each participant individually and explain their values and personal connection to the community and the study. Investigators also emphasized the history and purpose of specific medications (Table 6). As an additional layer of safety for participants, the principal investigators noted how they monitor each participant closely for adverse reactions, even more so than would be done outside of a research setting to further ease participants’ concerns. Participants have responded well to these efforts, and community members on the webinars felt these were helpful techniques, as they noted the importance of communication that is culturally appropriate.

**Table 6:** Barriers and solutions to clinical trial diversity.

Theme	Barriers	Solutions
**Access**	Transportation • Clinical research clinic sites located far from potential participants	• Transportation vouchers for participants to get to the research visit locations at no cost • Putting research clinic locations in the communities of populations of focus for recruitment
	Caregiver Burden • The role of caregiver for their family members and inability to take time away from caregiving	• Providing or paying for childcare and eldercare for participants
	Work • The type of employment doesn’t allow for taking time off for research visits during the day	• Extending clinic hours to outside of traditional business hours • Having visits early morning, in the evening, or on weekends
	Opportunity Cost • Participating in research may preclude other income generating activities	• Providing financial compensation for participation
**Awareness**	Contact to the Healthcare System • URM groups are less likely to have primary care and more likely to utilize the Emergency Department	• Disseminating information about trials through multiple, consistent and relevant channels to specific populations
	Language • Research staff unable to effectively communicate details about clinical trials available due to language barrier	• Using translators to communicate effectively with non-English speakers about trials • Translating recruitment materials into native languages of potential participants
	Health Literacy • Difficulty understanding details of health may preclude understanding of clinical trials	• Educating patients about their diagnosis and general health to allow for informed decisions about trial participation
	Research Literacy • Individuals may not know where to learn about or sign up for clinical trials	• Utilize community members as “research ambassadors” to educate communities consistent with “Community Scientist Academy” models
**Racism**	Interpersonal • Discrimination and racism by medical and research staff to participants • History of past medical abuses against racial/ethnic minorities cause lack of trust of healthcare institutions (also institutional & structural)	• Bias, discrimination, and anti-racism training for healthcare providers and clinical trial workforce • Working with each participant to explain direct benefits of a study to the participant with attention to cultural humility
	Institutional • Disinvesting in certain neighborhoods based on payor mix • Policies that don’t advance diversity, equity and inclusion in clinical research	• Advancing anti-racist policies, practices and procedures in institutions • Investing in communities of populations of focus • Working with community organizations to ensure research studies address needs, values and goals of the community
	Structural • Minority populations receiving lower quality of care and therefore having worse health outcomes	• Advancing anti-racist policies, practices and procedures across institutions
**Workforce Diversity**	Lack of Coordinator Diversity • Not having racially and ethnically diverse research coordinators, who are the primary recruiters for clinical trials	• Hiring coordinators and research staff from racially and ethnically diverse backgrounds as one avenue to better engage with racial/ethnic minority groups
	Lack of concordance • Investigators and research staff not racially, linguistically, or culturally concordant with the groups they are recruiting	• Cultural competence and humility from the research staff when engaging with their populations of focus • Working with medical providers in the communities of focus to have a direct pathway to recruiting those communities

Another strategy for addressing mistrust in minority populations is education [62]. Working with trusted community leaders and organizations to educate populations will increase the level of trust in the community. Additionally, disseminating and sharing study results directly with the communities that participated in the research and are impacted by the conditions the research is addressing will increase their engagement with the research field and make them feel like a valued part of the research process, which will also increase trust [63].

Furthermore, work discussing racism needs to be published. Some studies examine disparities between racial and ethnic minority groups in response to medications or treatments, but more needs to be done specifically to address racism in all forms from the socio-political lens, particularly within healthcare and in the communities healthcare organizations serve. Leading medical journals and organizations have recently begun to focus on the health effects and negative consequences of structural racism [64, 65]. The medical and research community must aggressively address racism if we wish to gain trust from marginalized groups.

While creating a trusting environment and relationship with racial/ethnic minority groups and the research workforce is essential, blaming low participation on racial/ethnic minority groups themselves is incorrect and must be curtailed. Framing the discussion of solutions must focus on eliminating the causes of mistrust and addressing the systemic racism that racial/ethnic minority populations face in order to gain trust [66]. As stated by Boyd et al., race should be used intentionally and for the right reasons when designing research studies. Not only should race and the reason for its inclusion be specifically identified and defined, but studies examining race should also include discussions on how racism affects health outcomes [66]. Dr. Boyd’s comments also speak to the need to measure racism in healthcare, and researchers and investigators in the webinar shared tools and strategies for measurement [59].

Webinar series attendees focused heavily on tackling issues of racism to increase clinical trial diversity in the dialogue with presenters and in the breakout room discussions. Many novel insights were garnered from those discussions. First, increasing researchers’ awareness of some minority groups being sensitive to the fear of identification due to undocumented status is key [67]. Attendees also discussed the importance of education, but specifically in educating the workforce on historical trauma faced by racial/ethnic minority populations so that they can engage from a place of understanding and humility. Attendees also discussed the idea of putting specific enrollment targets in place for White vs. non-White participants based on disease prevalence in the population, to promote targeted clinical trial diversity relevant to the phenotype of the disease.

### Clinical trial and primary care provider workforce diversity

The lack of diversity in the clinical trial workforce is another major barrier to increasing minority participation [68, 69]. Specifically, the lack of diversity in clinical research coordinators is a significant hurdle to performing high-quality, generalizable research for underrepresented racial/ethnic minority groups in medicine and science [20]. Clinical research coordinators are a key pillar in the successful recruitment and retention of participants in clinical trials, and diversification of the clinical research coordinator workforce has been a cornerstone of recent recommendations on solutions to diversify clinical trials [5, 70]. The investigator and clinical research coordinator are both vital to implementing inclusive trials, as coordinators are the face of the trial from the participants’ perspectives and principal investigators are typically the lead of the whole operation [5]. Cultural competence and humility by the principal investigator, coordinators, and site staff are essential for increasing clinical trial participation [5].

Studies show that racial concordance plays an important role in racial/ethnic minority groups’ decision to participate in clinical trials [69]. Therefore, having fewer investigators and research staff who are racial/ethnic minorities likely leads to less recruitment of minorities into trials [71, 72]. Racial concordance is helpful in racial/ethnic minority recruitment, but it is only the start of the researcher-participant relationship. Research staff must also have concordance in the aspects of language, culture, and goals for the community to truly engage and recruit racial/ethnic minority groups into clinical trials [62, 68]. Miscommunication causes misunderstandings of the details of a trial and can lead to hesitancy about participation, which is why it is important that researchers can understand the cultural and language barriers some populations may have [28]. Cultural beliefs on the part of the research staff are also important because it influences whom they seek for participation. Some providers and researchers believe that racial/ethnic minority groups are not interested in clinical trials, therefore they do not recruit them for their studies [62].

Another strategy for enhancing concordance and communication with racial/ethnic minorities in the literature includes establishing collaborations with primary care providers in the communities of interest [63]. Working through providers who have established trust with their current patients can lead to better understanding of the trial and benefits to potential participants. Another issue investigators have seen in study recruitment is patients being recruited based on a study qualifying condition who were not aware of their condition at the time of contact. This situation is why working with primary care providers is critical to not only obtaining permission to recruit patients, but to also ensuring patients are seeing their physicians and are aware of all their health issues.

All of these solutions may be effective in promoting diversity in clinical trial research participants, but are mere Band-Aids on the fundamental issue of a lack of diversity in the clinical research workforce. Minority researchers and scientists are more likely to recruit minority populations into their studies, but they make up a small portion of the research workforce [73]. Furthermore, the research topics proposed by racial and ethnic minority workforce populations are less likely to receive funding, yet another factor decreasing diversity in the clinical trial workforce and ultimately clinical trials [74]. Funding organizations must promote diverse researchers and research projects that focus on diverse communities in order to increase racial/ethnic minority participation in clinical trials. Luckily, these organizations are moving in the right direction, with the National Institutes of Health (NIH) creating grants and programs to increase funding for health disparity and community-based research. Additionally, programs like the Endocrine Society’s Future Leaders Advancing Research in Endocrinology (FLARE) program supports underrepresented individuals pursuing research and science by promoting their careers in endocrinology-related research [75]. Projects that create solutions to increasing the diversity of the research workforce will lead to an increase in the diversity of the participants as well.

### Strengths and limitations

There are many strengths to be highlighted with this work. The webinars were inclusive of multiple perspectives from individuals in academia, community members, clinician researchers, biotechnology and biopharmaceutical representatives, and employees or representatives of government organizations. By bringing together so many different groups, we were able to gain a rich diversity of perspectives. We also had a large number of participants across the two webinars, expanding the breadth of perspectives we were able to reach. Lastly, the development of all the topics and themes for the webinars were conducted with community member input to ensure we covered topics that were relevant to those we wish to serve.

Even with these strengths, our work must be considered in light of some limitations. First, a majority of the participants came from Ohio due to the seminars being hosted by an Ohio university and therefore generating the most interest from the surrounding area. Additionally, the webinars were held at noon during the weekday, which likely prevented some people who may have had interest from participating due to work-related commitments. By nature, group sessions may not allow for deep exploration of a topic from the viewpoint of one individual like an interview, and some people may not have shared sensitive information in the communal session; however, group discussions allowed us to obtain many perspectives in the webinar session. Lastly, we collected limited sociodemographic information from attendees, which we plan to enhance in future work.

### Conclusion

Clinical research has advanced medical treatments and interventions significantly over the past 50 years, but racial/ethnic minority groups are not receiving the same benefits as non-Hispanic White populations, and continue to experience disparities in chronic diseases and life expectancy [76]. Despite 41% of the U.S. population consisting of racial/ethnic minority individuals, this diversity is not represented in the clinical trial participant population [77]. This is a major issue for health and healthcare equity. Racial/ethnic minority groups are not to blame for this inequity. Rather, the cause is a lack of effective recruitment strategies addressing the significant barriers to clinical trial diversity. An intentional focus on solutions to the major barriers of access, awareness, discrimination and racism, and workforce diversity will improve recruitment and retention of racial/ethnic minorities in clinical trials and eventually lead to better health for the US as a whole. Hope is on the horizon as these disparities have been improving [3]; and COVID-19 clinical trials were more representative of the US population compared to prior studies [78, 79]. More funding organizations, like the NIH, are becoming intentional about promoting inclusive excellence [73, 80]; for instance, their large scale *All of Us* Research Program has paid particular attention to ensuring diversity from historically underrepresented groups [81], and the Food and Drug Administration provided guidance to the industry on enhancing clinical trial diversity [82]. Addressing the barriers and implementing the solutions identified through the “Health Equity through Diversity Webinar Series” and in the current literature are critical to sustainably advancing clinical trial diversity.

## Supporting information

S1 File Data for manuscript “Community engagement and clinical trial diversity: Navigating barriers and co-designing solutions—A report from the “Health Equity through Diversity” seminar series”. (PDF)
