# Supplementary material for: Community engagement and clinical trial diversity: Navigating barriers and co-designing solutions—A report from the “Health Equity through Diversity” seminar series
Source: PLoS One. 2023 Feb 16;18(2):e0281940. doi: 10.1371/journal.pone.0281940 (PMC9934412; doi:10.1371/journal.pone.0281940)
Supplement: S1 File — (PDF) [file pone.0281940.s001.pdf]

Supplemental File 1. Data for Manuscript "Community Engagement and Clinical Trial Diversity: Navigating Barriers and Co-Designing Solutions - A Report from the "Health Equity through Diversity" Seminar Series"

Scribe Notes from Moderated Breakout Sessions in the "Clinical Trials and Underrepresented Minorities: Mistrust, Misconceptions, Missed Opportunities and Moving Forward to Enhance Diversity" Webinar

The Breakout Sessions addressed 2 Questions:

Currently with all the great studies and science, only 5% of clinical trial participants are Black or African American and only 1% of clinical trial participants are Hispanic or Latino Origin.

1. What are the missing pieces?

2. What are potential solutions to increase these numbers?

- Missing pieces: We really need to mobilize and work on the solutions. We need to the barriers and we know some of the best practices. Bringing all the stakeholders together and align on solutions. There are great partnership opportunities. We spend a lot of time talking about barriers. We need to move focusing on solutions: education, awareness, breaking down barriers of mistrust or financial, engaging with communities. Ensuring sustainability.
- Missing pieces: There are many different companies doing the same thing. Why can't we work together to accomplish the goals. Let's put a list together. Let's get a state of Ohio List and work together to mobilize.
- Missing pieces: Meeting interest with digestible information. Be transparent. Feed communities information. Acknowledge historical traumas.
- Overcoming mistrust. Understand differences between populations. Getting buy-in from communities. Getting communities to become cheerleaders for this. Changing cultural in clinics. Having a champion in the clinics.
- Solutions – Engaging our community members as champions of clinical trials. Improving access to care and utilization of care. Cost of clinical trials (transportation, time off, etc). Collaboration opportunities are monumental – bring stakeholders together for financial support, grant opportunities, K-12 STEM programs.

- Lack of proper outreach, lack of connecting with the community
- From experience of a project coordinator in research – no actual research on recruiting black young and black adults in general. No studies done on mobilizing Black women either. How to come up with NEW recruitment strategies to reach these people?
- Wording, imaging, general advertisement of outreach and recruitment materials

- Remote recruitment is all through email and phone, but some recruitment really NEEDS to be in person in the community – faith organizations, rural organizations are hard to reach so you need to go to them.
- Think about LOCATION and where the recruitment is happening?
- Barriers to care (especially oncology) is COST. Many people won't even seek care because of the cost – even when they have insurance. This is a barrier to research because then they won't be recruited through their PCP
- Sex – some people prefer to speak with doctors of same sex. Same with race / other background, so engaging diverse PCPs
- Competitiveness – there can be competition between recruitment sites which can lead to staff recruiting however many people as possible and not focusing on diversity just on who they can get
- Some sponsors now have certain goals for % of diverse participants – from experience one member stated a trial she is working on has a CAP of white participants so that they can focus on recruiting AA. More sponsors/trials should consider doing this as it is a pretty new concept.

- There is a lack of minority primary care physicians. This is an especially large problem in lower-income areas. These PCPs have a lot of patients which may complicate forming deeper relationships with their patients. Increasing the number of minority PCPs will help both sides because it will allow PCPs and patients to form deeper relationships.
- There is a long history of experimentation by medical institutions in minority communities. The majority of people are aware of the Tuskegee experiments but many are unaware of similar trials in Hispanics and Puerto Ricans. Being aware of history is important. There may be a lack of education for physicians about this history.
- There isn't enough emphasis on diversity in the basic requirements of clinical trials. Right now there aren't stringent guidelines on what percentage of trial participants have to be minorities. Hardcoded rules surrounding this are missing in clinical trials.
- Minority groups want physicians that look like them but there are currently not enough. Increasing the number of minority physicians and healthcare providers is important.
- With the AZN trial, there were a lot of Caucasian people applying at the beginning. After 6 weeks, they got a message that they wanted a lot more diversity. They stopped enrolling Caucasians below the age of 65. After that, they were able to get more minority participants into the trial. Limiting the number of Caucasians in trials could be a step in the right direction.
- Information needs to be directly disseminated from physician to patient in their native language. In general, there is respect for physicians in Hispanic communities. If the information is coming from the physicians and the information being provided in Spanish, it may improve trial participation.

- People will not seek out trials if they afraid of giving identifying information if they are undocumented. Even those that are documented there may still be a fear of ICE. Overall, people need to trust the people that they are giving information. This starts with receiving information from institutions and people representing institutions that they trust. This would mean getting information directly from physicians, church leaders, community event organizers (i.e. in the downtown Latin festival).
- When OSU was trying to get the word out for the vaccination campaign they developed relationships with Community Vaccine Partners. These community stakeholders were churches, social service centers, or other organizations (i.e. the Urban League). Working with them allowed our information to be communicated by these institutions on our behalf. This worked with the vaccination campaign and so this strategy could be applied to clinical trials.
- There are technological barriers to sign-ups. Maybe asking for just a name and phone number instead of requiring email can help connect with more people. Being able to move along sign-ups with less information can lead to increased diversity.

- Reasons why people do/don't participate in trials
- Unmeasurable changes
- African American mistrust: representation/education
- Student & faculty involvement

- Major themes addressed were the under currents of mistrust between populations that hampered patient recruitment due to the political climate. Representation matters and if one does not see outside the orbit then there is lack of interest. Also, due to the lack of access of information to certain populations whether they are hurdles that are financial or based in logistics rely heavily on whether a study is accessible.
- Solutions proposed included multiple exposures to information about studies. Availability of staff permitting research only during business hours (labs closed, fedex hours) are rate limiting factors but there can be workarounds. Partnerships to avoid replicating efforts and not working in silos but have the neighbors, friends, families and not just companies work together to bring equity to healthcare and research.
- why are these populations so different from each other, the people who know about and won't participate and the people who aren't aware from the very beginning? People just didn't go get the care they needed therefore not aware
- clinical trial not hand in hand with clinical care
- "I want my insulin but I don't know whether to pay my food bill, electric bill"
- "if internet access is not present then no telehealth visit"

- not easy to access, find out about the clinical trial, email distribution lists and then what to do with information? How can this reach others?
- I propose multiple exposures, barrage of information but keep repeating the stimuli
- Representation, multiple exposures, conversations through trust
- Consistency is key in order for participants to be involved, versatility of hours, ease of access to location of research study
- Staff, safety, labs closed, FedEx hours are rate limiting factors but there can be work around
- Volunteered for trial and all for promoting science so not even thinking of why someone wouldn't want to participate or trust
- Area where partnership is important, no need to replicate efforts, not working in silos but have the neighbors, friends, families and not just companies
- We all don't think the same, I don't have qualms of participants, people on your team of different viewpoints, widening a circle of input, widening the issues where the issues aren't seen but definitely exist
- Racial minority, religious minority – perception and not automatically categorize or profile a person, situation, with representation to achieve health equity

- Gaining trust in the community
- Widening the net/ increasing the outreach efforts
- Striving for a more diverse research professional population. One where participants see professionals that look like them.
- Visit local community centers in diverse areas
- Work with community/church leaders to speak on health wellness opportunities
- Publishing results/success of research studies to increase motivation
- Consumer advisory groups to help design study & recruitment
- Using the music industry/producers to change the perception of research

- Understanding barriers
- Designing trials that account for those barriers and work to remove barriers in the trial process: ex. Transportation, parking, childcare for participants, making it easy for patients.
- Large barrier remains regarding familiarity and understanding clinical research in general
- Added education in community about clinical research/trials
- Including early education on scientific research
- Training future practitioners and clinical researchers to educate people in the community

- “Train the trainer”: Make sure those designing and conducting the research are educated about inclusive research methods and community engagement
- Education within clinician-patient relationship, since trust typically exists there already
- Education on population health and cultural factors in general to personalize the message about clinical trials
- In addition to racial background, consider age when designing approach to reach audience (ex. Social media vs. billboard, broadcast)
- Family approach when reaching community—involve the family in education and participation!!
- Budget for incentives and be creative in the marketing/recruitment plan of research projects--allocate sufficient funds for material/monetary incentives. Incentives work!
- Barriers such as a PCP providing information about clinical trials, mentality of “don’t go to the doctor unless you’re sick”
- Structural issues: implementing community outreach, navigation programs via health centers, understanding the needs of an area, “we need to provide the community with what they need, not what we think they need”
- Providing many avenues to reach out to a community and vice versa
- From a researcher: We recruit within clinic patient pool and therefore don’t see much diversity
- Potential benefit of positive experiences and sharing personal stories, especially when the storyteller looks like “me”
    - People can be influenced by those they look up to and respect
- More diversity within healthcare can have a “trickle down” effect to participants as well
- Seeing how the study can impact/benefit them and their friends, family, etc
- Helping educate staff – Help them understand potential backgrounds of fear and mistrust that may come from patients/participants
- Potentially providing compensation for the participant’s time
- Building a relationship with the community you are looking to engage
    - Long-term relationship! Not last-minute, but long-standing
- Spreading the word via different avenues and models
- Showing direct benefits of past clinical trials, seeing how science has helped people in the past
    - What is standard of care now, we know because of past clinical trials
- Would you participate in a trial, and if so, only a specific type? (We had some extra time)
    - One woman and her daughter have participated in clinical trials (eye studies)
        - Curiosity and altruistic nature were the primary incentives
- Yes definitely, including a drug trial, because wide representation is so important
- **We learned from our moderator about the different phases of a clinical trial when testing on humans (such as the COVID 19 vaccines)

- There is a lack of exposure and advertisement within the neighborhoods of groups lacking involvement. In addition, clinicians within a given community may lack the resources to inform potential participants about clinical studies.
- Doctors may operate based on the assumption that a patient will not be interested in the clinical trial. Similarly, the clinician may hesitate to ask patients if they are interested in a clinical trial if there are language barriers present that make it difficult to explain the clinical trial adequately.
- The COVID-19 pandemic may have an effect on patients' willingness to participate in a clinical trial.
- There is a distrust in communities of color in regards to medical research and experimental trials.
- Recruitment should be tailored to the targeted group so that the trial is culturally interesting. This would require the use of focus groups that reflect the desired community in order to learn what they would like to see in regards to the best places to recruit, the best way to alleviate transportation difficulties, and how to have a culturally relevant approach/ relevant incentives.
- Building meaningful relationships with community leaders and black medical societies would help build trust between the community and healthcare and research institutions. This trust is a key component of increasing involvement.
- Educate patients about the disease state and the direct significance of the trial for their community. Help patients understand why their participation is critical to equitable health care and better outcomes for their community.

- Regardless of care, seem great need of training of culture competence. Recruiter should be trained for competence health literacy needs a great update.
- Lack of resources, people don't know where to go. Either PI recruiting patients, also patient contact people in the community. Lack resources also stop PI from recruiter of minorities as well as language barrier.
- Where u go? How to connect w/ community? The academic institution is different from community and takes years to develop the connection/relationships. While this may not be what PI want, took a lot relational work and this is not just science.
- How to balance the resources and language barrier? – find the resources can help with those only offer study in English language. More people actively looking for translators. Bring in company help with translation.
- Trying to do bilingual studies. All about what you have done in your studies and share it with other PIs. Build up resources.
- Train RA/coordinators to work

- Health care system different in different countries
- How you feel when things go virtual during th pandemic – how that effect the participant?
- COVID bring the awareness to the equity
- Also, people were unable to come that they don't have / couldn't find a child care. And other various challenging. People don't want to come other's home even though they were willing to pay for homecare.
- Differences between research and the actual health care

- Lack of knowledge. We don't know about all of the different trials. If there was more of a digital/social media push, we would be able to reach the younger generation better.
- A lot of the information out there are not as "digestible". It could be very intimidating to patients. During the vaccine trial, they placed individuals in a room with a large consent form. Reading this by yourself could be intimidating. We should encourage healthcare providers to personalize their engagements with patients. Individuals with no medical knowledge or background should be properly guided through the respective process.
- We need to find all of the barriers on each level (physician, pharmacy, etc.) that are preventing people from connecting with trials. Next, we should begin forming solutions and personalize each response under a specific lens.
- Youth, specifically students, should help explain clinical trials to parents, senior citizens, or anyone who is not aware of the potential trials available.
- Encourage public outreach campaigns to help bridge the gap between trials and eligible patients.
- Question - Are there reimbursements for patients: Paying for parking, providing child care, covering costs for missed time at work, etc.? Is there a national standard? Does this become incentivized? Is this considering inducing patients?
- Use social media as a platform to reach the younger generation
- Social media will not reach the older generation, so we need to tailor how we connect with individuals who are not on Instagram, Facebook, etc.
- People who have been enrolled in clinical trials should share their own experiences with others. This will help spread awareness, build trust, and establish connections among the minority communities – specifically, those who are hesitant in enrolling in a trial.

- Representation of diverse researchers is important to attract diverse participants
- If we had that representation, it could increase the number of people of color in clinical trials
- Do you think there is a stigma around clinical trials and vaccine programs?
- Having more diverse researchers would definitely increase the number of diverse participants

- How do we encourage people to participate in clinical trials- as a clinical researcher she takes time and effort to let the participants know that she is genuinely interested in their health to build that trust? Coming from a place of humility helps to develop trust and ultimately increase participation
- Having a spirit of humility and treating participants the same way you would like to be treated
- How do we increase the numbers to get a much more diverse population to participate in clinical trials?
    - Compensating people for their time, or for transportation
    - Treat the families as a whole, so that the participant can be free to do what is required of them e.g. Having someone watch the kids when the participant is needed, bring your significant other to help read through all the paper work and to answer any questions they may both have.
    - Making participants feel valued because we are the people who need the research done, so it is important the help them in any way we can to make it easy for them to participate e.g. Parking costs, transportation

- What are some of the ways we are using to include more diverse populations?
    - Using platforms such as Google to recruit participants and also share what trials are recruiting participants
    - This has to also come from the community-so it is important to involve community members as we plan clinical trials
    - We also need to share the importance of the clinical trials especially with a number of trials such as Alzheimer's which we are not participating in, because donated brains are actually needed to help advance the understanding of this disease.
    - Word of mouth is also quite powerful especially when a participant has had a great experience, so we need to make sure that the experience of the clinical trials are good

- Language matter- operation warp speed implied that the vaccine production was too quick to be safe
- Cost of health care may deter people from accessing healthcare to be able to be recruited for clinical trials
- The expansion of telemedicine may offer additional recruitment opportunities
- Telemedicine allows patients to be seen in their own territory and seeing part of their world and may help them to feel more comfortable- and gives an opportunity to ask the provider questions about the vaccine, clinical trials, etc.

- Community members not having enough access to credible information and the opportunity read the science(professionals can access PubMed, journals etc.) delivered by people who look like them from the community
- Addressing health equities in developing countries and countries with fewer resources (example-current COVID-19 crisis in India)
- Having more people in the field to contact potential participants
- Increasing public confidence by highlighting the success of the clinical trials
- Having more diversity among clinical trials staff and professionals
- Innovation: There was an example discussed where a company was able to send out portable EKG monitors instead of patients coming to the office for an EKG due to COVID-19. Maybe these types of strategies would be feasible for reaching the communities

- Barriers:
  - Lack of Awareness
  - Economic Issues
  - Communication
  - Mistrust
- Language/Communication Barriers – rapport building is missing through devices and interpreters
- Pharmaceutical companies we go to well-populated areas, need more reach out to underserved clinical settings. From Chicago Suburbs – Trying to reach the underserved population in Chicago. Trying to get investigators that look like the patients. Like with Rush University and Cook County (Charity Hospitals).
- From the Industry perspective, the topic is unfamiliar at her company. The first step at her company is to start measuring and establishing metrics.
- The term minorities as a noun, we need to move towards being specific. Denotes a lesser than perspective.
- What sites are underserved groups accessing? Do we go to the community colleges? How do we reach people through social media?
- How do we get beyond churches and barbershops? How do we approach these things? What about the people who aren't showing up.
- In regards to Social Media, it can be hard to know what is legitimate. Personalized approaches vs. automated if using social media.

- Solutions:
  - pretrial Planning
  - Patient Education
  - Commitment
  - Concern from study staff

- Financial Incentives
- Word of Mouth
- Increased Clinical Trial Diversity in the Workforce
- Incentivizing companies to increase clinical trial diversity with increased patent time
- Have the providers speak to their patients.
- Community Champions to peak interests and introduce study to individuals
- Social Media is age dependent and using trusted stakeholders in the community.
- New Media Solutions like Clubhouse.
  Include participants and community advocates from the beginning
- Office of Community Engagement within UNC Cancer Center
- Increasing awareness in the communities, going to where the people are

- Increase accrual through their doctors
- Strategies – recruit our patients through our clinic and research is part of their treatment
- We would go out to events that are related to the cultures that we are trying to engage with in terms of research. We would also talk about what research is which we found to be important.
- You need the people.
- What about the group that is sharing the information, does that matter?
- Who should place it on social media, should it be community organizations?
- Linguistic Concordance – important for retention. Cultural Concordance
- Humility is important.
- Mistrust vs. Distrust
- Celebrities
    - Speakers – Two very tough questions. No Silver Bullet.
    - Accepting our seat at the table for underrepresented communities.
    - Having a voice.
    - TRUST
    - Education on both sides – consumer and practitioner side. Train the trainer approach on the clinician side.
    - Loved the diversity of perspective. This was not healthcare people talking to healthcare people.
    - Balance of perspectives
    - What brought you to this forum? Need an ice breaker.

Scribe Notes from Moderated Breakout Sessions in the "Community Engagement: Strategies for Cultivating and Sustaining Meaningful Partnerships: Scribe Notes from Breakout Sessions" Webinar

The Breakout Sessions addressed the following question:

"How can we drive meaningful partnerships to advance health among community members and stakeholders with healthcare orgs, industry, and government?"

Moderators could address the question by asking:

What brought you here today as a participant of this webinar?

What does meaningful partnership mean to you?

What was the impact and outcomes from the partnership (for those who had participated in previous partnerships)?

- We need to be very specific in the requests when reaching out to individual partners – can always have people who are general partners but need to come in with very specific requests, so they know what they are dealing with and know whether these requests are within their abilities
- Come into the communities and interact with the partners – get exposed to personal relationships before reaching out to make a request
- Get people from all different backgrounds with different specialties to interact with the communities
- Important to bring the right people to the table – understand who the movers and shakers are – it is even more important sometimes to know who is not at the table so we can get them involved
- Make sure that people are aware of the purpose or bigger picture of the community, stakeholder, health care organizations, industry, and government partnerships when they are interacting

What brought you here today as a participant of this webinar?

- Being able to partner with other groups to reduce fetal/infant mortality.
- To learn more about community-based research.
- To help others learn more about clinical trials and how they important they are.
- To be an advocate for diversity in clinical trials.

What does meaningful partnership mean to you?

- You are accountable for your part of the partnership. You need to hold up your end of the partnership and hold your partners accountable.
- Everybody has a piece to add. No single organization can do it by themselves.

- Keep focused and work together for success.

What was the impact and outcomes from the partnership?

- Have seen amazing impact in the Black Impact 100 project.
- Clinical trials changes lives!
- In many of the projects we heard about today we can see immediate impact – we don't' have to wait until the next generation.
- You can't make changes in the community without having the community involved from the start.

How do you drive meaningful partnerships?

- Accountability, connecting with people.
- Implement the results – put them into action and drive policy changes.
- Lessons learned
- High blood pressure is still an issue and we need more research to find effective ways to lower it. BP should be a focal point.
- Make surveys short and easy to complete.

Why everyone joined today's webinar:

- Interested in the webinar due to background in public/population health
- Pharmaceutical/clinical trial backgrounds and a desire to learn more about the webinar series
- Collaborator with Dr. Gray, staying up to date with goings on at OSU and initiatives
- What do meaningful partnerships mean to you?
- Honesty, importance of meeting people where they are, "borrowed trust"
- Having an open dialogue of goals, hopes, and for the stakeholder to then provide honest feedback
- How can goals be approved? What elements are being forgotten? Asking questions and discussing to create a more complete and impactful plan
- Discussing can also help find common ground for people who are possibly on opposite ends of the spectrum
- How can we drive meaningful partnerships to advance health?
- Connect with someone locally from the company you're wanting to partner with—it can take time to find the right person in the company, but starting locally helps get that process started
- Being willing to invest the time needed to make the partnership successful
- Really listen to the other side's needs and history
- Allow the community to also lead
- Let community leaders lead!
- Involving the community allows for very valuable and accurate feedback + identification of a community's need(s)

- Have you engaged in any meaningful partnerships in the last 5 years?
- Pfizer and OSU
- Able to engage around colorectal screening and providing outreach to minority groups
- Northwestern
- Trying to build community partnerships through outreach and support of their existing community partners
- Giving them more tools to address specific community needs
- Merck (?)
    - Partnered with advocacy groups
    - Would like to do more outreach with faith-based groups (underutilized avenue for them currently)

Impact and Outcome of Partnerships – Ran out of time.

What brought you here today?

- Keeping it simple and clear and don't promise what we can't deliver
- How to make connections with community or management to work with the patient population; how to get through the gatekeepers
- I would like to learn from the community and the gatekeepers what I need to do to assure that I understand and know how to meet their needs. I have the desire but recognize that I do not have a complete understanding of this community. Willing to learn

- How can we drive meaningful partnerships to advance health among community members and stakeholders with health care organizations, industry, and government?
- Partnerships allow for deep and careful planning that can benefit the community
- COVID-19: As a hospital system we did not have a great outreach to give back to the community, however, the pandemic allowed us to look at specific locations and see where certain areas were hurting more than others. We then were able to reach out to these specific areas to help out, leverage partnerships, and understanding community needs
- We know that these issues need to be addressed and having resources/partnerships are extremely important to helping the community
- Personal stories are great tools to rooting and setting the foundation of great relationships.
- Risks/Barriers to meaningful partnerships:
- Getting bogged down into broad and bigger issues in community engagement

- Being able to understand different areas or neighborhoods uniquely help with case based approaches which may allow for better results

- Lack of trust, developing patience with developing trust, listening to what communities need and listening to what the communities themselves have to say
- Some people may be scared to even see a doctor, so echoing that trust within these communities is very important
- Taking the time to understand communities and the nuances of each community is necessary

- Inclusion more than just getting people in study – inclusion to actually gather meaningful data and results
- Community education activities – precursor to meaningful relationships
- Science cafes in the evenings to educate people – open forum for community to ask questions about research, what they think research is, do they trust it, believe in it?
- Help better understand research better
- Foundation to build trust in the medical profession
- No presentations – just and open forum, open communication
- Trying to understand patient population, needs of the health system
- Help community affected (by rare disease) understand specifics about the disease
- Doing education specific to orgs based on the population they serve
- Building trust by expressing that Pfizer's goals are the same as the organization they are helping
- Focus on meeting needs of individual customers
- Coming to listen at the beginning is very important
- Setting & showing your priorities and goals up front
- Communicate to understand where different groups are coming from
- Don't go in just trying to fulfil YOUR study – a meaningful partnership includes recognizing what the community needs and focusing on addressing those issues/problems/needs
- Adding value for the community / population
- You cannot open a site and just expect patients to come – they are scattered. Need to partner with local orgs / communities to reach out to the patients

How can we drive meaningful partnerships to advance health among community members and stakeholders with healthcare organizations, industry and government?

- Everyone introduced themselves
- What do meaningful partnerships mean in relation to partnering with other organizations?
- Partnerships are key, without them you cannot reach the communities for lasting change and to decrease the lack of access and improve preventions. From Natalie's experience when organizations come together, they have been able to meet community needs, unlike if they worked independently
- Partnerships also bring together funding opportunities to help and serve the communities in the best way possible

- Denise also mentioned that her mentor always said that you have to build relationships to establish trust to result in getting the work needed done
- E.g walking and knocking on doors in the community, meeting people where they are comfortable ore where they are at
- Be persistent and consistent and be visible at local business areas
- Be inclusive and don't forget our pharmacies for access to medicines after patients have been diagnosed
- Pharma's need to better partner with communities

How can we drive meaningful partnerships to advance health among community members and stakeholders with health care organizations, industry, and government?

- Know what the community hopes to gain (should be the main basis for any discussion)
- Without this knowledge it is difficult to provide value on both sides
- Representation matters and relationships with community leaders gets someone trusted, who shares the interest of the community, involved with the process
- Health screenings and accessibility (health fairs and health education)
- Health literacy (that understands and meets problems surrounding language barriers, etc.)
- Important to meet people where they are (understand their attitudes surrounding their health and other factors that impact those decisions/health behaviors)
- Find areas that connections can be more easily facilitated
    - i.e., barbershops, basketball tournaments, radio stations, community social media pages
- Utilizing borrowed trust
- working with trusted organizations already established in communities and supporting the work they already do

- Building partnership requires time investment and building trust.
- People to experience something and then describe what they experience. Model after that and in turn give someone else the opportunity to also experience that. Hands on setting (home type). Cooking, eating and dialoging to promote the change you want to see in the community. Work with and learn from the people impacted every day by these disparities. Community members often have suggested solutions. Always asks "what would you like to see". Also explain what opportunities there are that they may not know of already.
- What kind of partnerships it is effects type of engagement and outreach. Requires genuineness and seek of understanding, what the needs and wants are that can be provided. mutual respect
- Have you engaged in any meaningful partnerships in the last 5 years?

- Pharmaceutical representative felt it was important to bring different pharmaceutical perspectives. Recognized “blind spots” so it's important to take in different perspectives and promote health equity. “it's one thing to know how to do it, when do to, and do it fast, but its another thing to bring other people along” “understand where people are and focus on the outcome because the process may change” “understand the community's total picture, and if you don't understand, be humble enough to ask”

- What brought you here today as a participant of this webinar?
- Involved in Tobacco related project and comes to learn and share information.
- Works with a community partner on food justice. Seeking information to stay equipped to how to talk to the community about vaccinations and total health.
- In pharmaceutical industry, how to best understand performances and how to create results that are wanted/needed. Here to listen to different thoughts and views
- Here to report back to Executive Director of Minority Health

- Meaningful Partnerships
- Imperative to eliminate disparities and promote Health Equity
- Community partner with local organization seeks to get more involved in health equity and has enjoyed the benefits of prior collaborations.
- Organizations that want to partner need to do community assessments to see where the need is
- Organizations that are partnering to have frequent contact with those communities not just a monthly visit
- Bring the voices to the fore front of those being impacted
- What does the community need not just what we think they need. Do not make an assumption but “peel back the onion”
- Strategies on how to make an impact in these communities

What does meaningful partnership mean?

- Collaborate
- Agree to disagree, we do not always think or see the same
- Maintain the goal of decreasing disparities and increasing Health Equity
- The four C’s, Communicate, Collaborate, Coordinate and Connectivity
- Listening skills-we need to listen
- Two way partnership, helping those not of black or brown communities understand the disparities.
- We can do this together

- We have a long way to go.
- We spend the most on healthcare and have the shortest life expectancy.
- Girlfriend is DACA -. The impact of BIPOC COVID-19 -> girlfriend had COVID, which has been tough, we have to improve the systems.
- What does meaningful partnership mean?
- Having a space in the community, need to have roots in the clinic. Disinvestment in communities of color has had a major impact.
- Foundations within in the community to partner with.
- Putting effort in, convincing people that it’s worth the investment. With Latino populations will you end up on the radar of ICE, developing trust. You have to hear the fears and address them, so that you can have a more transparent relationship.
- Budget for the public health vs. the police – important to discuss. Safe communities has different connotations based on who you are.
- Building trust, engage the schools and police dept. I work at taco bell and you need to come see me during our lunch break. Time is a major factor and time of day for partnerships is important.
- Not everyone has the ability to have access to transportation and babysitting.
- working at the Franklinton Farm – discussed the importance of direct community engagement
- Having the same language is important.
